# Clinical Characterization of [^18^F]T-008, a Cholesterol 24-Hydroxylase PET Ligand: Dosimetry, Kinetic Modeling, Variability, and Soticlestat Occupancy

**DOI:** 10.2967/jnumed.123.265912

**Published:** 2023-12

**Authors:** Cristian C. Constantinescu, Terry Brown, Shining Wang, Wei Yin, Olivier Barret, Danna Jennings, Johannes Tauscher

**Affiliations:** 1 Invicro, New Haven, Connecticut; and; 2 Takeda Pharmaceutical Co. Ltd., Cambridge, Massachusetts

**Keywords:** soticlestat, cholesterol 24-hydroxylase, dose occupancy, PET imaging, developmental and epileptic encephalopathy

## Abstract

This series of studies characterized [^18^F]T-008, a PET radiotracer for imaging cholesterol 24-hydroxylase (CH24H), in healthy volunteers (ClinicalTrials.gov identifier NCT02497235). Assessments included radiation dosimetry, kinetic modeling, test–retest variability (TRT) evaluation, and a dose occupancy evaluation using soticlestat, a selective CH24H inhibitor. Soticlestat is currently in phase 3 development for the treatment of seizures in Dravet syndrome and Lennox–Gastaut syndrome. **Methods:** In the dosimetry study, 5 participants (3 men) underwent serial whole-body scans to estimate organ-absorbed doses and effective doses of [^18^F]T-008 using OLINDA/EXM 1.1. For the kinetic modeling and TRT study, 6 participants (all men) underwent two 210-min dynamic [^18^F]T-008 PET scans with arterial blood sampling. The regional total volume of distribution was estimated using a 1-tissue-compartment model, a 2-tissue-compartment model, and Logan graphic analysis. In the dose occupancy study, 11 participants (all men) underwent 120-min scans at baseline and 2 time points (peak and trough) after receiving single oral doses of soticlestat (50–600 mg). The relationship between effect-site soticlestat concentration and brain occupancy was evaluated with a specially developed pharmacokinetic model and a saturable maximal occupancy model. **Results:** The estimated mean whole-body effective dose was 0.0292 mSv/MBq (SD, 0.00147 mSv/MBq). [^18^F]T-008 entered the brain rapidly, with a distribution consistent with known CH24H distribution densities. The 2-tissue-compartment model and Logan graphic analysis best described the tracer kinetics. The mean TRT for estimating total volume of distribution was 7%–15%. Single doses of soticlestat in the range 50–600 mg resulted in occupancies of 64%–96% at 2 h and 11%–79% at 24 h. The estimated half-maximal effect-site concentration of soticlestat was 5.52 ng/mL. **Conclusion:** [^18^F]T-008 is a suitable PET radiotracer for quantitatively analyzing CH24H in the human brain. Using [^18^F]T-008 and PET, we demonstrated that soticlestat was brain-penetrant and established target engagement by displacing [^18^F]T-008 in a dose-dependent manner in the brain.

## Visual Abstract

**Figure:**
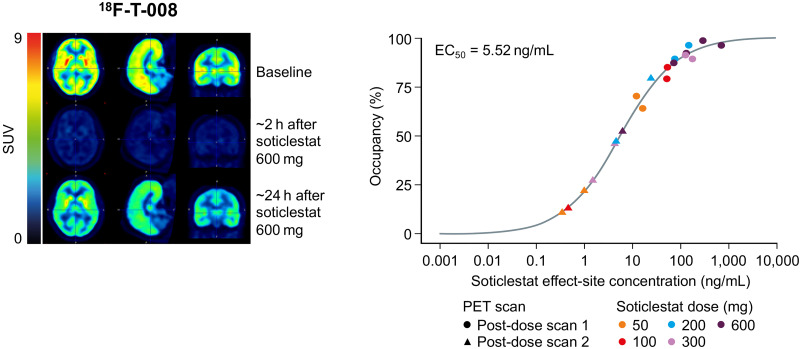

Cholesterol homeostasis in the brain is a highly regulated process that is crucial for normal neuronal integrity and function (1*,*^2^), with its dysregulation implicated in epilepsy, stroke, and neurodegenerative diseases (3*,*^4^). Neuronal damage can result in the release of cholesterol from neuronal cell membranes, increasing cholesterol concentration in the local environment (5*,*^6^).

The blood–brain barrier prevents direct transportation of cholesterol out of the central nervous system. Consequently, the major pathway for the elimination of cholesterol from the brain is via conversion of cholesterol to 24*S*-hydroxycholesterol (24HC), which can cross the blood–brain barrier (1). Cholesterol 24-hydroxylase (CH24H, also known as CYP46A1) is selectively expressed in the brain and is responsible for catabolizing cholesterol to 24HC (1). 24HC has been shown to positively modulate the function of *N*-methyl-d-aspartate glutamate receptors, which have been implicated in the pathophysiology of several neurologic disorders, including epilepsy (7*,*^8^).

The availability of a PET ligand that targets CH24H would enable evaluation of the pathophysiologic role of CH24H in neurologic conditions in vivo and aid clinical dose selection for novel drug therapies that target cholesterol metabolism. Soticlestat is a selective CH24H inhibitor (9) that is currently in phase 3 development for the treatment of the developmental and epileptic encephalopathies Dravet syndrome and Lennox–Gastaut syndrome (ClinicalTrials.gov identifiers NCT04940624 and NCT04938427, respectively).

Here, we report a series of clinical imaging studies with [^18^F]T-008, a PET imaging radiotracer for CH24H that has previously demonstrated high selectivity and specificity in nonhuman primates (10). The studies reported here include dosimetry of [^18^F]T-008, tracer kinetic modeling with test–retest variability (TRT) assessment, and evaluation of the effect of pharmacologic doses of soticlestat on in vivo binding of [^18^F]T-008 in the brain (ClinicalTrials.gov identifier NCT02497235).

## MATERIALS AND METHODS

### Radiosynthesis

The radiolabeling and preparation of [^18^F]T-008 (also known as [^18^F]MNI-792) were performed according to the procedures described in U.S. Food and Drug Administration Investigational New Drug application 123966, using a commercial synthesizer, TRACERlab FX-FN (GE Healthcare). Details are provided in the supplemental materials (available at http://jnm.snmjournals.org).

### Study Objectives

There were 3 primary objectives: to determine the whole-body distribution and radiation dosimetry of [^18^F]T-008, to model the tissue-specific kinetics of [^18^F]T-008 in the brain, and to establish target occupancy of soticlestat using [^18^F]T-008 and PET.

### Study Design and Participants

This open-label, phase 1 study was conducted on healthy volunteers. Individuals of either sex were eligible for inclusion if they were aged 18–50 y (dosimetry and test–retest studies) or if they were aged 19–55 y, weighed at least 45 kg, and had a body mass index of 18.0–30.0 kg/m^2^ (occupancy study). Additionally, participants for all studies had to be in good health (as determined by physical, electrocardiography, and laboratory test evaluations). Volunteers were excluded if they had a positive urine or other test result for drugs of abuse, alcohol, or the nicotine metabolite cotinine at screening or admission for imaging visits. Women with a positive pregnancy test result (dosimetry and test–retest studies) or of childbearing potential (occupancy study) were also excluded.

For each participant, before injection of [^18^F]T-008, vital signs (blood pressure, heart rate, respiratory rate, and oral temperature), electrocardiography results, and results from clinical laboratory tests (serum chemistry, complete blood count, and urinalysis) were assessed.

Study protocols were reviewed and approved by the New England Institutional Review Board. All participants gave written informed consent before participation in the study.

### Safety and Tolerability

Safety and tolerability assessments were conducted at all study visits for the dosimetry and test–retest studies, and included laboratory tests, electrocardiography, physical and neurologic examinations, and recording of adverse events.

### Whole-Body Distribution and Dosimetry

Participants received an intravenous bolus [^18^F]T-008 dose of 340.2 ± 14.9 MBq (9.2 ± 0.4 mCi). Directly afterward they underwent PET (ECAT EXACT HR+; Siemens): 9 whole-body passes were performed in succession, up to approximately 5.5 h (2 × 60, 3 × 120, 4 × 270 s/bed position and two 45-min breaks), with scans from the tip of the head to the thighs at 9 bed positions. Urine samples were collected after the fifth, seventh, and ninth passes.

Methods for data acquisition and calculation of residence time (total number of disintegrations) in source organs are presented in more detail in the supplemental materials (11–14).

### Brain Kinetic Modeling and TRT Study

#### Image Acquisition and Processing

Details of the image acquisition and reconstruction parameters are given in the supplemental materials. After a 3-min intravenous bolus injection of [^18^F]T-008 at a dose of 326 ± 37 MBq (8.8 ± 1.0 mCi), dynamic PET scans were acquired over 3.5 h including a 30-min break after the first 90 min. Before any PET activities, a 3-dimensional T1-weighted MR image was acquired for each participant for anatomic identification and image processing. Image processing was performed with PMOD 3.405 (PMOD Technologies). The probabilistic volume-of-interest atlas (11) was applied to normalized [^18^F]T-008 images, and time–activity curves were extracted from major brain regions.

#### Kinetic Modeling

Arterial blood samples were collected throughout the imaging period and analyzed for [^18^F]T-008. A subset was processed by reversed-phase high-performance liquid chromatography to estimate the fraction of intact (unmetabolized) [^18^F]T-008 and was used to obtain an arterial input function (AIF) for kinetic modeling. The plasma free fraction (f_P_) was measured by ultrafiltration.

Kinetic modeling was performed with 1-tissue-compartment (1TC) and 2-tissue-compartment (2TC) models and using Logan graphic analysis (LGA) with a cutoff linear regression equilibration time of 30 min. The total volume of distribution (V_T_) was then estimated using the metabolite-corrected plasma AIF. Goodness of fit was evaluated using the Akaike information criterion (a smaller Akaike information criterion indicates a more appropriate model). The nondisplaceable binding potential (BP_ND_) was derived indirectly from the V_T_ and the nondisplaceable V_T_ (V_ND_): BP_ND_ = (V_T_ − V_ND_)/V_ND_. In this equation, V_ND_ was the V_T_ of the cerebellum, considered a reference because of its low density of CH24H (15). The BP_ND_ was also estimated directly by noninvasive modeling (i.e., not requiring any blood data) using the noninvasive version of LGA (NI-LGA) (16) and by the simplified reference tissue model (SRTM) (17) using the cerebellum as a reference. For the NI-LGA, the cutoff linear regression equilibration time was fixed at 30 min, and the efflux rate constant of the reference region (*k*_2_′) was fixed at the value obtained from the SRTM, fitting to data in the putamen (the region with the highest signal).

#### TRT

The TRT of the different outcome measures was assessed for each analysis method as TRT (%) = 100 × (test − retest)/[(test + retest)/2]. Reliability was evaluated by assessing the intraclass correlation coefficient (ICC), defined as (between-participant mean square − within-participant mean square)/(between-participant mean square + within-participant mean square).

### Soticlestat Occupancy Study

#### PET Scans

Each participant received a baseline scan and 2 postsoticlestat (blocking) scans on days 1 and 2, respectively. Data were acquired dynamically over 120 min, an interval based on time-stability analysis of data from the kinetic modeling and TRT study.

#### Soticlestat Administration

The first 2 participants received a single dose of soticlestat, 600 mg, as per the protocol. For each dosing cohort, a dose-level review meeting occurred to review the imaging occupancy results and safety data. The dose level and timing of the imaging were adjusted accordingly during the study.

#### Assessment of Occupancy

Arterial blood was collected during the baseline and first blocking scans. The weighted average [^18^F]T-008 metabolite-corrected AIF from these scans was used to model the AIF for the second blocking scan. Weights were based on the injected [^18^F]T-008 doses (IDs), as follows:

$${\text{AIF}}^{\text{blocking}\;2}\;=\;{\text{ID}}^{\text{blocking}\;2}\times \left({\text{AIF}}^{\text{baseline}}/{\text{ID}}^{\text{baseline}}+{\text{AIF}}^{\text{blocking}\;1}/{\text{ID}}^{\text{blocking}\;1}\right)/2.$$

V_T_ was estimated using LGA because this method is less sensitive to the input function shape and was, therefore, preferred over the 2TC model. Brain CH24H occupancy was estimated directly from Lassen occupancy plots (18):

$${\text{V}}_{\text{T}}{}^{\text{baseline}}-{\text{V}}_{\text{T}}{}^{\text{blocking}}\;=\;\text{occupancy}\times \left({\text{V}}_{\text{T}}{}^{\text{baseline}}-{\text{V}}_{\text{ND}}\right).$$

Occupancy is the slope of the plot, and V_ND_ is the *x*-intercept.

The BP_ND_ was estimated from the V_T_ using the participant’s V_ND_ estimated at highest occupancy (first blocking scan), as follows:

$${\text{BP}}_{\text{ND}}\;=\;{\text{V}}_{\text{T}}/{\text{V}}_{\text{ND}}-1.$$

Regional occupancy estimates were obtained from BP_ND_ at baseline and after soticlestat dosing, as follows:

$$\text{Occupancy}\;\left(\%\right)\;=\;100\times \left(1-{\text{BP}}_{\text{ND}}{}^{\text{blocking}}/{\text{BP}}_{\text{ND}}{}^{\text{baseline}}\right).$$

#### Soticlestat Pharmacokinetics and Pharmacodynamics

During both blocking scans, serial blood samples were collected immediately after tracer injection (t = 0) and at 1 h and 2 h after tracer injection for determination of plasma concentrations of soticlestat and its *N*-oxide metabolite. Serial blood samples were also collected at the following time points for pharmacodynamic analysis of plasma 24HC levels: 1, 4, and 12 h after check-in on day −1 (day before the first blocking scan); before dosing (≤30 min before dosing) and 1, 4, 8, and 12 h after soticlestat administration on day 1; and 20, 24, and 30 h after soticlestat administration on day 2.

Plasma concentrations of soticlestat, its *N*-oxide metabolite, and 24HC were measured using high-performance liquid chromatography with tandem mass spectrometry detection. The assay was validated over a concentration range of 1–2,000 ng/mL for soticlestat and its *N*-oxide metabolite, and 2–100 ng/mL for 24HC.

#### Relationship Between Soticlestat Plasma Concentration and Occupancy

A population pharmacokinetic model for soticlestat was developed empirically by combining the plasma-concentration data from the current study with those collected from single- and multiple-rising dose studies (19*,*^20^). A model linking predicted plasma soticlestat concentrations (from the population pharmacokinetic model) with brain occupancy values determined from PET imaging and the model-predicted concentration at the time of PET tracer injection was initially developed. However, temporal discrepancies between plasma drug concentration and brain occupancy values were observed, suggesting a lack of equilibrium at the effect site. Consequently, an effect-site compartment model was developed (21), linking predicted soticlestat concentrations at the effect site with brain occupancy values (22).

The relationship between plasma concentration and brain occupancy was modeled with a saturable sigmoidal maximal occupancy (E_max_) model:

$$\text{Occupancy}\;\left(\text{\%}\right)\;=\;\frac{{\text{E}}_{\text{max}}C^{\text{γ}}}{\left({\text{EC}}_{50}^{\text{γ}}+C^{\text{γ}}\right)}\times 100.$$

E_max_ is the predicted maximal occupancy, EC_50_ is the predicted half-maximal effective concentration, and γ is a sigmoidal shape factor. Concentration, *C,* was either the plasma concentration measured from samples collected in the PET study or effect-site concentration obtained from modeling. For the relationship between occupancy and plasma concentration, γ was fixed as 1. For the relationship between occupancy and effect-site concentration, γ was obtained from nonlinear fit. E_max_ was fixed at 100%.

## RESULTS

### Participants

The study included 22 healthy volunteers. The overall mean age was 41 y (SD, 7 y) (range, 25–51 y), and the mean body weight was 85.4 kg (SD, 11.4 kg) (range, 58.2–101.6 kg). Participants received a mean tracer activity of 332.2 MBq (SD, 27.9 MBq) (range, 215.6–358.6 MBq), with a corresponding mean injected mass of 1.13 μg (SD, 0.66 μg) (range, 0.36–3.00 μg) and mean molar activity of 139.6 GBq/μmol (SD, 74.8 GBq/μmol) (range, 39.4–342.3 GBq/μmol). Per-participant data are summarized in Supplemental Table 1.

Five participants (3 men, 2 women) with a mean age of 42 y (SD, 7 y) (range, 35–49 y) underwent whole-body PET imaging. Six male participants (mean age, 43 y [SD, 6 y]; range, 32–48 y) underwent 2 PET scans, 3–21 d between each scan, for assessment of TRT. Eleven healthy male participants (mean age, 38 y [SD, 8 y]; range, 24–50 y) completed the soticlestat dose occupancy study.

### Safety Data

No clinically significant adverse findings in clinical laboratory parameters, vital signs, electrocardiograms, or physical examinations were identified during the study. Details of adverse events are provided in the supplemental materials.

### Whole-Body Distribution and Dosimetry

**FIGURE 1.**
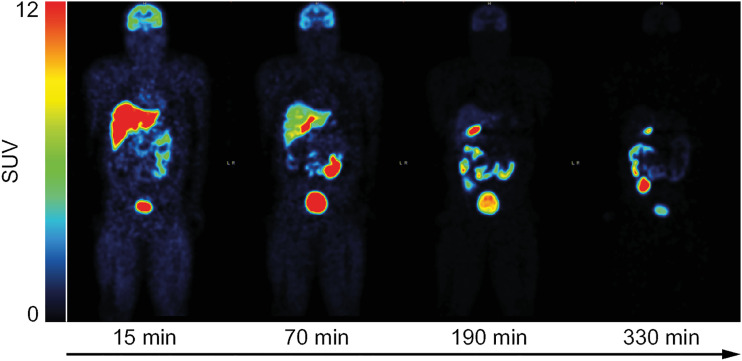
[^18^F]T-008 whole-body coronal PET images showing tracer distribution change over 5.5 h in healthy participant (participant M3; Supplemental Table 1).

Whole-body coronal PET images over time for participant M3 are shown in Figure 1. These images illustrate that the tracer was eliminated via both hepatobiliary and urinary routes. Brain, gallbladder, heart, intestines, kidneys, liver, and urinary bladder were designated as source organs (activity above background in at least 1 acquisition frame; Supplemental Table 2; Supplemental Fig. 1). The overall mean maximum uptake, as percentage injected dose, was 20.0% (SD, 8.4%) in the liver and 33.5% (SD, 5.1%) in the intestines. The mean total cumulative radioactivity present in urine, as percentage injected dose, was 38.6% (SD, 1.1%) (Supplemental Table 3).

Overall mean absorbed doses and whole-body effective dose are summarized in Table 1. The gallbladder absorbed the highest radiation dose (mean, 0.206 mSv/MBq [SD, 0.0989 mSv/MBq]), identifying it as the critical organ. The estimated mean effective dose was 0.0292 mSv/MBq (SD, 0.00147 mSv/MBq). Additional [^18^F]T-008 whole-body distribution data are provided in the supplemental materials.

**TABLE 1.** Mean Absorbed Doses and Whole-Body Effective Dose After Injection of [^18^F]T-008

Target organ	Mean dose (mSv/MBq)
Adrenal glands	1.07E−02 (2.01E−03)
Brain	2.77E−02 (5.45E−03)
Breasts	4.36E−03 (1.08E−03)
Gallbladder wall	2.06E−01 (9.89E−02)
Heart wall	1.05E−02 (1.91E−03)
Kidneys	2.28E−02 (8.12E−03)
Liver	4.22E−02 (9.01E−03)
Lower large intestine wall	5.14E−02 (2.28E−03)
Lungs	6.17E−03 (1.44E−03)
Muscle	8.45E−03 (1.23E−03)
Osteogenic cells	9.49E−03 (2.13E−03)
Ovaries	2.95E−02 (1.59E−03)
Pancreas	1.29E−02 (2.22E−03)
Red marrow	9.91E−03 (1.02E−03)
Skin	4.87E−03 (9.55E−04)
Small intestine	1.31E−01 (7.91E−03)
Spleen	8.16E−03 (1.62E−03)
Stomach	1.23E−02 (1.59E−03)
Testes	6.45E−03 (8.49E−04)
Thymus	5.08E−03 (1.38E−03)
Thyroid	4.43E−03 (1.02E−03)
Upper large intestine	1.47E−01 (1.11E−02)
Urinary bladder	1.52E−01 (3.30E−02)
Uterus	2.81E−02 (1.93E−03)
Total body	1.15E−02 (1.32E−03)
Effective dose*	2.92E−02 (1.47E−03)

* Based on International Commission on Radiological Protection Publication 60 (13).

Data in parentheses are SD.

### Brain Kinetic Modeling and TRT Study

[^18^F]T-008 readily entered the human brain and was ubiquitously distributed with high to moderate accumulation in the putamen; caudate nucleus; nucleus accumbens; pallidum; thalamus; frontal, occipital, parietal, insular, and temporal lobes; anterior and posterior cingulate; amygdala; and hippocampus. Accumulation was low in the brain stem and cerebellum. Peak uptake, expressed as SUV, was approximately 8 in the putamen about 30 min after injection. Washout was much faster in the cerebellum, in which uptake peaked up to 5 min after injection.

**FIGURE 2.**
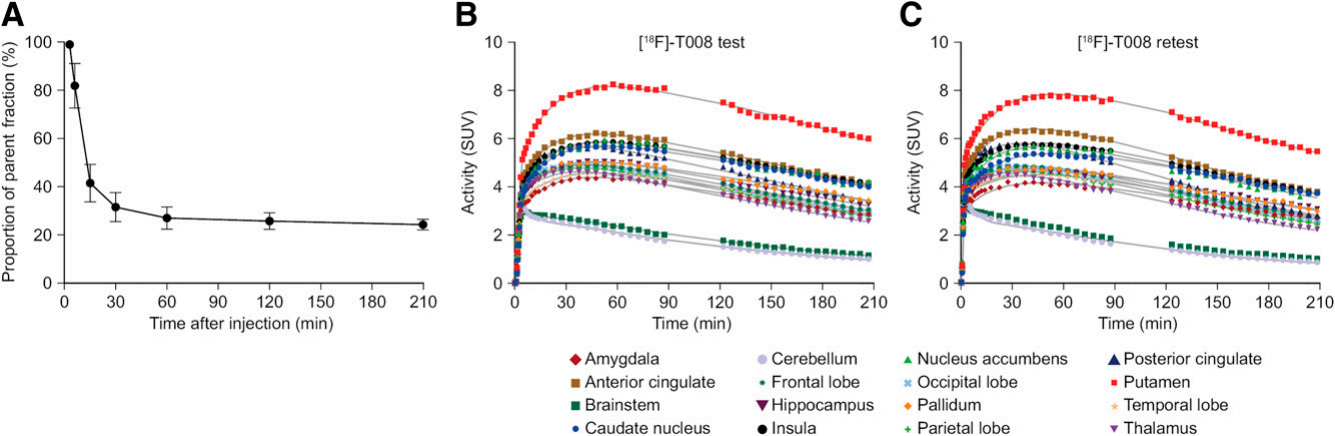
(A) Mean percentage of unmetabolized [^18^F]T-008 for participants enrolled in test–retest study. (B and C) Representative regional brain time–activity curves after bolus injection of [^18^F]T-008. Data are SUV for healthy human participant after bolus injection of [^18^F]T-008 for test (B) and retest (C) parts of study. Gaps in curves correspond to break periods (time out of camera). Continuous lines represent 2TC model curves.

[^18^F]T-008 was rapidly metabolized. Up to 27% of the parent compound remained at 60 min after injection. Subsequently, the parent fraction reached a plateau that was maintained at approximately 25% up to 210 min (Fig. 2A). The mean f_P_ of the parent compound in plasma (i.e., not protein bound) was estimated to be 48% (SE, 3%; *n =* 12).

Representative mean test–retest time–activity curves from a single participant, averaged over 210 min, are presented in Figures 2B and 2C. On the basis of the Akaike information criterion, the 2TC model was better suited than the 1TC model to describe the data (Supplemental Fig. 2). V_T_ and BP_ND_ values were estimated using either 210 min or 90 min of data to evaluate the effect of reducing the imaging time. Mean V_T_ values, estimated with 2TC and LGA using 210 min of data, in participants in the test cohort are summarized in Supplemental Table 4. Mean BP_ND_ values, estimated with 2TC, LGA, and NI-LGA (with the cerebellum as the reference region) using 210 min of data, in participants in the test cohort are summarized in Supplemental Table 5.

**FIGURE 3.**
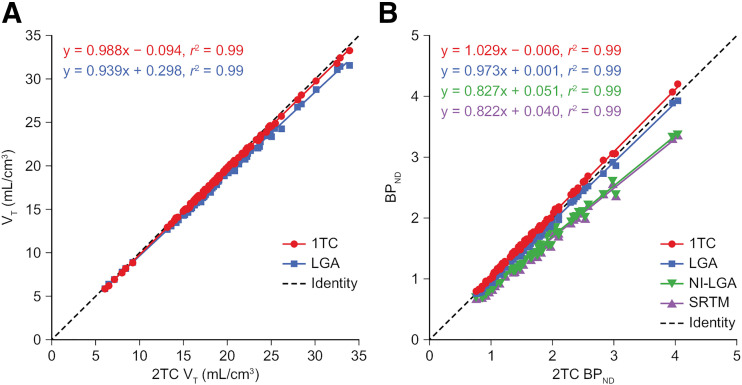
Correlations between 2TC V_T_ and 1TC and LGA estimates (A) and between 2TC BP_ND_ and 1TC, LGA, NI-LGA, and SRTM estimates (B). All estimates were obtained by modeling 210 min of imaging data from first scan of TRT study. Dashed line in each graph is line of identity; solid lines show linear regression.

Correlations between V_T_ and BP_ND_ obtained with the 2TC model, 1TC model, LGA, SRTM, and NI-LGA are provided in Figure 3. V_T_ and BP_ND_ estimates derived using LGA and 1TC were highly correlated with those derived using 2TC, close to the line of identity, with *r*^2^ being 0.99 (Fig. 3). SRTM and NI-LGA underestimated BP_ND_ by approximately 15% compared with 2TC (Fig. 3B). The mean absolute TRT and ICC values across the 6 participants in the test–retest cohort are presented in Supplemental Table 6 for V_T_ and in Table 2 for BP_ND_. Mean absolute TRT values were 7%–15% for V_T_ and 3%–15% for BP_ND_. TRT was similar across all methods used. ICC values were consistently high for BP_ND_ across all brain regions, indicating high reliability. In contrast, ICC values for V_T_ varied substantially across the examined brain regions. The ICC values were generally positive, indicating that variance was mainly due to between-participant differences rather than within-participant differences. Similar absolute TRT and ICC values were estimated for V_T_ and BP_ND_ across different models.

**TABLE 2.** Absolute TRT and Intraclass Correlation Coefficient for BP_ND_ Estimated With 1TC and 2TC Models, LGA, NI-LGA, and SRTM

	Mean absolute TRT (%)					Intraclass correlation coefficient				
Brain region	1TC	2TC	LGA	NI-LGA*	SRTM*	1TC	2TC	LGA	NI-LGA*	SRTM*
Amygdala	6 (5)	7 (6)	6 (7)	6 (6)	6 (5)	0.95	0.94	0.94	0.96	0.96
Anterior cingulate	6 (7)	6 (7)	6 (6)	6 (5)	5 (5)	0.91	0.89	0.92	0.95	0.95
Caudate nucleus	5 (4)	6 (5)	6 (4)	5 (3)	5 (4)	0.97	0.97	0.96	0.98	0.98
Frontal lobe	4 (3)	5 (4)	4 (4)	4 (4)	4 (4)	0.95	0.93	0.92	0.95	0.95
Hippocampus	11 (6)	12 (6)	11 (7)	9 (6)	10 (6)	0.92	0.90	0.91	0.94	0.94
Insula	5 (4)	6 (5)	5 (4)	3 (3)	4 (3)	0.95	0.94	0.95	0.98	0.98
Nucleus accumbens	9 (7)	9 (7)	8 (6)	6 (5)	7 (5)	0.92	0.90	0.91	0.94	0.94
Occipital lobe	7 (5)	8 (5)	7 (5)	6 (4)	6 (4)	0.85	0.85	0.86	0.91	0.91
Pallidum	15 (12)	15 (12)	15 (11)	12 (10)	12 (9)	0.82	0.81	0.83	0.89	0.89
Parietal lobe	5 (4)	6 (5)	6 (5)	5 (4)	5 (4)	0.92	0.91	0.91	0.94	0.94
Posterior cingulate	5 (3)	6 (3)	5 (4)	4 (4)	4 (4)	0.82	0.76	0.78	0.86	0.86
Putamen	5 (3)	5 (4)	4 (4)	3 (2)	3 (2)	0.97	0.96	0.98	0.99	0.99
Temporal lobe	6 (4)	6 (5)	6 (4)	5 (4)	5 (4)	0.89	0.87	0.88	0.93	0.93
Thalamus	9 (7)	10 (9)	10 (9)	8 (6)	9 (6)	0.90	0.88	0.87	0.92	0.92

* Noninvasive method.

TRT was estimated using 210 min of data. Estimates with 1TC, 2TC, and LGA required plasma input. Data in parentheses are SD.

### Soticlestat Occupancy Study

The first 2 participants in this cohort (M12 and M13) received soticlestat, 600 mg, and were imaged at 0.75 and 10 h after dosing. On the basis of a review of data from this initial cohort, the timing of imaging for the remaining participants, who each received a soticlestat dose of 50, 100, 200, 300, or 600 mg, was changed to 2 h (peak) and 24 h (trough) after dosing. All soticlestat doses used and the timing of [^18^F]T-008 PET imaging are presented in Supplemental Table 1.

**FIGURE 4.**
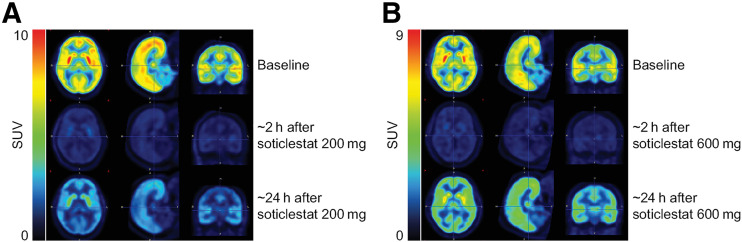
Representative brain images for baseline [^18^F]T-008 PET scan (top row) and postdose PET scans at 2 h (middle row) and 24 h (bottom row) after dosing with soticlestat, 200 mg (A) or 600 mg (B). Data are from healthy participants who received single dose of soticlestat.

Representative images (averaged over 120 min of acquisition) at baseline and at 2 and 24 h after a single dose of soticlestat, 200 or 600 mg, are presented in Figure 4. The decrease in intensity observed across all gray matter regions after soticlestat dosing versus baseline was related to the timing of PET acquisition relative to soticlestat dosing, as well as to the soticlestat dose.

The plasma metabolite profile of [^18^F]T-008 was similar to that observed in the TRT study, with a mean of 30% (SD, 8%) remaining at 60 min after injection, followed by a plateau at about 25% that was maintained up to 120 min. No major differences in [^18^F]T-008 metabolism between baseline and the first scan after soticlestat administration were observed. The mean f_P_ was 41% (SD, 4%) at baseline and 40% (SD, 3%) after soticlestat administration, with no significant effect of soticlestat on f_P_. Furthermore, no trend was observed for f_P_ as a function of soticlestat dose.

On the basis of the findings from the kinetic modeling study, LGA using AIF was used to calculate V_T_, with Lassen plots being used for estimation of global occupancy. V_T_ values are summarized in Supplemental Table 7. Single doses of soticlestat in the range 50–600 mg resulted in occupancies of 64%–96% at 2 h and 11%–79% at 24 h (Supplemental Table 1).

**FIGURE 5.**
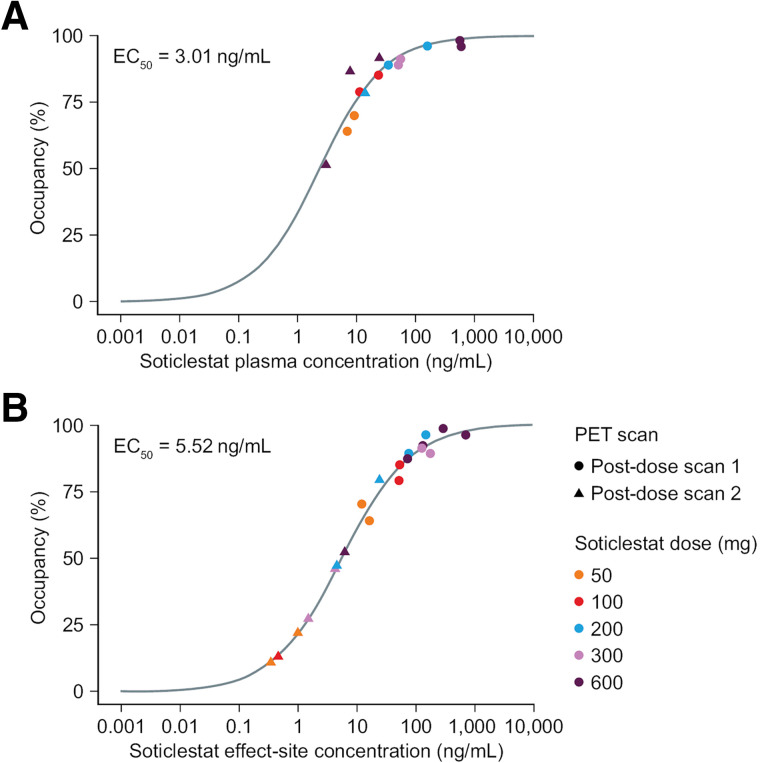
Brain CH24H occupancy estimates as function of soticlestat plasma concentration (A) and soticlestat effect-site concentration (B). Soticlestat plasma concentrations were measured from samples collected in PET study. Soticlestat effect-site concentrations were derived from population pharmacokinetic model. Sigmoid asymptotic maximum effect model was used to fit curve to data. Symbols and colors identify data corresponding to each single soticlestat dose and 2 postdose PET scans.

Plasma soticlestat and its *N*-oxide metabolite concentrations increased with increasing soticlestat dose. After soticlestat dosing, plasma 24HC concentrations decreased across the soticlestat dose range (50–600 mg). The relationship between both plasma concentration (measured) and effect-site concentration (modeled) and brain CH24H occupancy is shown in Figure 5. Plasma concentration values corresponding to the lower part of the occupancy spectrum were below the limit of quantification and could not be used to model the relationship with occupancy. Effect-site concentration values derived from the pharmacokinetic model were available at all occupancy values. The EC_50_ estimates for plasma and effect-site were 3.01 ng/mL (relative SE, 16.61%) and 5.52 ng/mL (relative SE, 10.80%), respectively. The global γ-value was 0.758 (relative SE, 3.2%). The parameters were generally estimated with good precision (Supplemental Table 8). The goodness-of-fit and visual predictive check plots for the concentration-occupancy model indicate that the model described the data reasonably well.

### Data Sharing

The datasets, including the redacted study protocol, redacted statistical analysis plan, and individual participants’ data supporting the results reported in this article will be available 3 mo after the submission of a request, to researchers who provide a methodologically sound proposal. The data will be provided after its deidentification, in compliance with applicable privacy laws, data protection, and requirements for consent and anonymization.

## DISCUSSION

We report the first—to our knowledge—in vivo human PET studies conducted to clinically validate [^18^F]T-008 as a CH24H-specific radioligand, based on biodistribution, reproducibility, and target specificity, and assessment of radiation dosimetry and pharmacokinetic parameters.

The biodistribution of [^18^F]T-008 showed that the tracer was cleared via both hepatobiliary and renal routes, with no organ presenting an abnormally high uptake. The critical organ was the gallbladder, with the gallbladder wall receiving the highest mean absorbed dose. Although still at an acceptable level, the dose received by the gallbladder can be reduced through the intake of food or drink with a high fat content immediately after the PET scan to stimulate bile ejection (23). The mean whole-body effective dose (0.0292 mSv/MBq) is comparable to the effective doses of other [^18^F]labeled PET ligands, which typically lie within the range 0.015–0.03 mSv/MBq (24–28).

[^18^F]T-008 distribution in the brain was consistent with our prior observations in the nonhuman primate brain and with CH24H densities observed in western-blotting analysis (10). Our findings also align with known CH24H messenger RNA expression in the human brain (29): ubiquitous expression, with the highest density observed in the basal ganglia regions and cerebral cortex. Lower densities are observed in the white matter, brain stem, and cerebellum. Furthermore, the regional distribution of V_T_ values reflected the distribution of tracer uptake expressed as SUVs.

Kinetic modeling results identified 2TC as the preferred model over 1TC for [^18^F]T-008 brain PET quantification. LGA V_T_ values were slightly lower than 2TC V_T_ values, which is in line with a report of noise-induced underestimation of V_T_ values using a graphic method (30). LGA was supported as an appropriate method to use for V_T_ estimation because it demonstrated low negative bias and variability compared with 2TC. Furthermore, LGA is more robust than 2TC with respect to the AIF, which, for the soticlestat dose occupancy study, allowed us to use an AIF based on AIFs from the first 2 scans for the third scan (second after dosing), thus avoiding excessive arterial blood draws.

TRT across participants (7%–15% for V_T_) was similar for all models used. Furthermore, time-stability analysis demonstrated that acquisition time could be as low as 90 min, with minimal impact on V_T_ bias and variability. Because TRT study time–activity curves did not include data from 90 to 120 min after injection, time stability could not be assessed with 120 min of dynamic data. However, for the dose occupancy study, we selected a scan time of 120 min to minimize bias and variability in V_T_ estimates.

Oral dosing with soticlestat resulted in substantial blocking in all brain regions, including the cerebellum, as is in line with the results in nonhuman primates (10). At high soticlestat occupancy (≥80%), the cerebellum V_T_ decreased by about 68% from baseline (from ∼6.4 mL/cm^3^ to ∼2.0 mL/cm^3^), which was slightly above the average V_ND_ of about 1.5 mL/cm^3^. Subsequently, these findings excluded the cerebellum as a candidate reference tissue for noninvasive quantification with [^18^F]T-008. As such, the V_ND_ could not be obtained directly from any region of reference. Therefore, both CH24H occupancy and V_ND_, when possible, were estimated directly from the occupancy plots on the basis of V_T_ only. In the absence of a reference region, BP_ND_ values were calculated indirectly on the basis of V_T_ and V_ND_ to allow for the estimation of regional occupancies, which presented a small SD. This calculation demonstrated that no brain region exhibited occupancy significantly different from the global occupancy estimates. Because V_ND_ could not be accurately determined for the 2 participants who received soticlestat, 50 mg, neither BP_ND_ nor the regional occupancies could be obtained. Missing regional occupancy data could explain the differences in the EC_50_ estimates when compared with the corresponding occupancy estimates, modeled from high-uptake regions such as the caudate or putamen.

Occupancy ranged from 11% at trough plasma levels to 98% at peak plasma levels. These measurements were both dose- and time-dependent, increasing with increasing dose and decreasing with time. Many of the pharmacokinetic samples collected during the dose occupancy study did not contain measurable levels of soticlestat, particularly at low occupancy values. Consequently, limited pharmacokinetic data were available and a pharmacokinetic model was developed to describe the complex nonlinear pharmacokinetics of soticlestat, using available data from single- and repeated-dose studies. The model was adequate in characterizing the pharmacokinetic data used in the analysis (22).

The model-predicted soticlestat concentrations were subsequently used to develop a concentration–occupancy model in which they were linked to brain occupancy values. A saturable E_max_ model that used the predicted effect-site soticlestat concentrations provided a better fit for the data because there appeared to be a temporal lag between plasma soticlestat concentrations and brain occupancy values at later time points. This lag suggests that the plasma drug concentration was not in equilibrium with that at the effect site, namely the site of action where the initial pharmacologic response is produced.

Interestingly, the EC_50_ value estimated with the predicted effect-site concentration (5.52 ng/mL) did not shift significantly (i.e., by more than an order of magnitude) from the EC_50_ value estimated with the plasma data (3.01 ng/mL) when using a simplified version of the E_max_ model (a sigmoidal shape factor, or Hill slope, was fixed as 1 by assuming noncooperative binding).

## CONCLUSION

Data reported here demonstrate that [^18^F]T-008 is a useful PET radiotracer for in vivo imaging of CH24H in the human brain. Kinetic modeling results indicated that the quantification of [^18^F]T-008 requires arterial sampling because no suitable reference region lacking CH24H could be found. The estimated mean effective dose suggests that an acceptably low radiation exposure is associated with [^18^F]T-008 imaging in human participants and is consistent with values reported for other PET radiotracers currently used in human studies (24–28). Soticlestat specifically engaged CH24H in the brain, generating a wide range of dose-dependent occupancy values. Additionally, its effect-site concentration relationship with occupancy was well described by a saturable E_max_ model. Findings from these studies were used to guide the schedule and dosing for clinical trials with soticlestat.

## DISCLOSURE

This study was funded by Takeda Pharmaceutical Co. Ltd. Terry Brown, Shining Wang, Wei Yin, and Johannes Tauscher are employees of Takeda Pharmaceutical Co. Ltd. and own stock or stock options. Cristian C. Constantinescu is an employee of Invicro, which was contracted by Takeda Pharmaceutical Co. Ltd. to conduct the study. Olivier Barret and Danna Jennings are former employees of Invicro. Under the direction of the authors and funded by Takeda Pharmaceutical Co. Ltd., Aimee Jones and Erin Aldera of Oxford PharmaGenesis, Oxford, U.K., provided medical writing support for this publication. Editorial assistance in formatting, proofreading, copyediting, and fact-checking was also provided by Oxford PharmaGenesis. No other potential conflict of interest relevant to this article was reported.

## Notes

KEY POINTS **QUESTION:** Is [^18^F]T-008 a suitable PET radiotracer for imaging CH24H in the human brain? **PERTINENT FINDINGS:** [^18^F]T-008 entered the brain rapidly, with a distribution consistent with known CH24H distribution densities. **IMPLICATIONS FOR PATIENT CARE:** The brain kinetic properties and dosimetry of [^18^F]T-008 provide the opportunity to foster global use of in vivo CH24H PET imaging in neuroscience research.
